# Enteral Nutrition within 48 Hours of Admission Improves Clinical Outcomes of Acute Pancreatitis by Reducing Complications: A Meta-Analysis

**DOI:** 10.1371/journal.pone.0064926

**Published:** 2013-06-06

**Authors:** Jie-Yao Li, Tao Yu, Guang-Cheng Chen, Yu-Hong Yuan, Wa Zhong, Li-Na Zhao, Qi-Kui Chen

**Affiliations:** Department of Gastroenterology, Sun Yat-Sen Memorial Hospital, Sun Yat-Sen University, Guangzhou, Guangdong, People’s Republic of China; University of Szeged, Hungary

## Abstract

**Background:**

Enteral nutrition is increasingly advocated in the treatment of acute pancreatitis, but its timing is still controversial. The aim of this meta-analysis was to find out the feasibility of early enteral nutrition within 48 hours of admission and its possible advantages.

**Methods and Findings:**

We searched PubMed, EMBASE Databases, Web of Science, the Cochrane library, and scholar.google.com for all the relevant articles about the effect of enteral nutrition initiated within 48 hours of admission on the clinical outcomes of acute pancreatitis from inception to December 2012. Eleven studies containing 775 patients with acute pancreatitis were analyzed. Results from a pooled analysis of all the studies demonstrated that early enteral nutrition was associated with significant reductions in all the infections as a whole (OR 0.38; 95%CI 0.21–0.68, *P*<0.05), in catheter-related septic complications (OR 0.26; 95%CI 0.11–0.58, *P*<0.05), in pancreatic infection (OR 0.49; 95%CI 0.31–0.78, *P*<0.05), in hyperglycemia (OR 0.24; 95%CI 0.11–0.52, *P*<0.05), in the length of hospitalization (mean difference −2.18; 95%CI −3.48−(−0.87); *P*<0.05), and in mortality (OR 0.31; 95%CI 0.14–0.71, *P*<0.05), but no difference was found in pulmonary complications (*P*>0.05). The stratified analysis based on the severity of disease revealed that, even in predicted severe or severe acute pancreatitis patients, early enteral nutrition still showed a protective power against all the infection complications as a whole, catheter-related septic complications, pancreatic infection complications, and organ failure that was only reported in the severe attack of the disease (all *P*<0.05).

**Conclusion:**

Enteral nutrition within 48 hours of admission is feasible and improves the clinical outcomes in acute pancreatitis as well as in predicted severe or severe acute pancreatitis by reducing complications**.**

## Introduction

Acute pancreatitis (AP) presents in about 80% of patients as a course without serious morbidities and with a low mortality rate [1]. But once organ failure (OF), which is thought to be one of the consequences of systematic inflammation response syndrome (SIRS), or infected pancreatic necrosis occurs, mortality raises from 3% to 30% and 32% respectively [2].

It is well demonstrated that the damage of gut barrier is responsible for the initiation of SIRS and sepsis and associated with the infected pancreatic necrosis. Gut barrier is damaged in the early phase of AP and intestinal permeability is significantly increased in severe attacks of AP within 72 hours [3]. As a consequence, translocation of the inflammation compounds and the toxic products from the gut occurs, which can lead to SIRS and OF [4]. Moreover, the bacterial flora in the intestine gains access to the systemic circulation through the damaged gut barrier, which causes sepsis or infected pancreatic necrosis in the very early phase of the disease. A study reported that infection of the pancreas increased from 33% in the first 24 hours to 75% between 48 and 96 hours with significant statistical difference [5]. Therefore, the maintenance of the gut barrier, as well as the timing to do so is crucial for the recovery of patients with AP.

AP accompanied with inflammatory response, toxic products, and infection is an energy consuming course, so how to provide nutrition to the AP patients has been studied for decades. In the beginning, total parenteral nutrition (TPN) was introduced, aiming at “pancreatic rest” and “gut rest”. But no advantages of TPN on the total hospital stay or incidences of complications of pancreatitis were detected. Besides, intestinal atrophy was noticed, making the condition even worse [6]–[8]. However, enteral nutrition (EN) was found to be better at maintaining the gut barrier by helping to modify the lactulose/mannitol ratio, lower the plasma endotoxin level, maintain the normal makeup and distribution of intestinal microbial, and lower the bacterial translocation [9]–[12]. In clinic practice, EN provided a safer route to feed patients with predicted severe acute pancreatitis (pSAP) or severe acute pancreatitis (SAP) than TPN, which was demonstrated by a meta-analysis [13]. But the timing to start EN was not emphasized in this analysis and it varied among different trials and different hospitals [14]–[16]. Though it is clear that long-lasting TPN or total “gut rest” brings no benefits, questions about whether there is no need for “gut rest” at all and whether EN rather than TPN is better in the very early phase of AP still bother a lot of physicians. In consideration of the early damage of gut barrier mentioned above, early EN initiation, namely, EN within 48 hours of admission, may bring more definite advantages.

Some recent researches compared early EN with late EN or TPN. In this study, a meta-analysis was conducted to further identify whether early EN could bring benefits to AP patients.

## Patients and Methods

### Search Strategies

We searched PubMed, EMBASE Databases, Web of Science, the Cochrane library, and scholar.google.com for all the relevant articles about early EN from inception to December 2012. Medical Subject Heading (MeSH) or key words as “enteral nutrition”, “nasojejunal”, “jejunal”, “nasogastric”, “tube feeding”, “parenteral nutrition”, “jejunostomy”, “ileostomy”, and “gastrostomy” were searched with “acute pancreatitis” using logical operator “and” respectively. Reference lists of all included articles were scrutinized to disclose additional literature on this topic.

### Selection Criteria

Studies that were included must fulfill the following criteria: (i) design: available randomized comparative trials (RCT) or retrospective comparative trials fully reported with detailed information; (ii) population: patients with AP; (iii) intervention: EN initiated within 48 hours of admission and controlled by TPN or EN outside 48 hours.

Studies were exclude if they were: (i) duplicate publications; (ii) case report, review, meta-analysis, or guideline; (iii) not reporting clinical relevant outcomes; (iv) not providing enough details.

### Data Extraction and Management

The following information was obtained from the included studies: the first author, year of publication, the starting time of EN, the severity of AP, the number of participants, the EN route, design features of the studies, the number of all the infections as a whole, catheter-related septic complications, pancreatic infection, hyperglycemia, pulmonary complication, OF, death, and the length of hospitalization (LOH) of both early EN group and the control group. Additionally, stratified analysis was conducted based on the severity of AP.

### Bias Assessment

The included studies were assessed for risk of bias by two independent researchers according to the Cochrane guidelines [17]. Individual methodological domains of the included studies reporting randomization sequence, allocation concealment, and blinding were graded accordingly: (i) adequate = methods were reported and appropriate; (ii) inadequate = methods were reported but inappropriate; or (iii) unclear = methods were not reported.

### Statistical Analysis

Statistical analyses were done by using the computer program Review Manager (Version5.1 for Windows, Cochrane Collaboration, Oxford, UK) chiefly and STATA (Version 12.0; STATA Corporation, College Station, TX, US) was used in the quantitative assessment of publication bias as supplement.

Pooled odds ratio (OR) and 95% confidence interval (CI) were calculated. The OR values of <1.0 represented an advantage for the early EN group compared with late EN or TPN group. The overall effect was considered to be significant at the 0.05 level. The *I* ^2^ test and *Q* test were used to evaluate statistical heterogeneity among the included studies. A value of *I* ^2^ measure more than 50% or a *P* value of the *Q* test lower than 0.10 was considered representative of statistically significant heterogeneity. A random-effect model was used in statistics with heterogeneity, and a fixed-effect model was used in statistics without heterogeneity. Furthermore, stratified analysis was conducted based on the severity of AP and the corresponding studies were assigned to either mild AP (MAP) subgroup or the pSAP or SAP subgroup. The OR, its 95% CI, and heterogeneity of either subgroup were calculated respectively. The subgroup differences were assessed and a *P* value of less than 0.05 was considered representative of statistically significant.

As for bias, a sensitivity analysis between RCTs and the retrospective comparative studies was performed. The effects of including retrospective studies on OR and the heterogeneity were assessed. The potential publication bias was evaluated and demonstrated by the Begg’s test and the Egger’s test with STATA quantitatively. A *Pr* or *P* value of less than 0.05 was considered representative of statistically significant publication bias.

## Results

**Figure 1:**
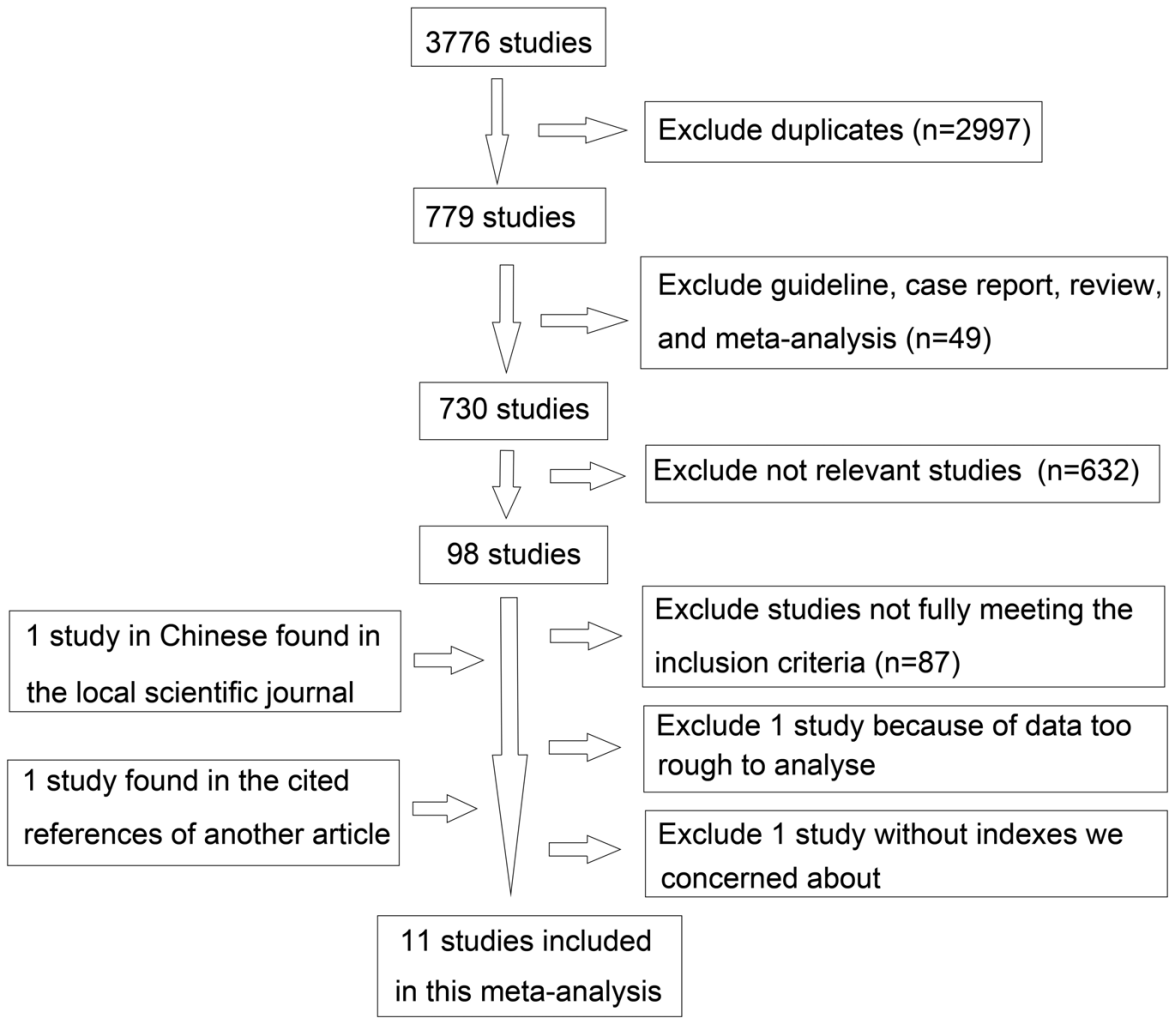
Flow chart of literature search for meta-analysis.

A total of 3,776 studies were retrieved from PubMed, EMBASE Databases, Web of Science, the Cochrane library, and scholar.google.com. After the duplicates were identified and excluded, 779 were left. Then we also excluded the case report, review, guideline, and meta-analysis according to the title or abstract, leaving 730 studies among which 98 were found to be relevant. We closely reviewed these 98 studies and excluded 89 articles. Among them, 87 were excluded because they did not fully meet the including criteria; one RCT was excluded because it was a PYTHON trial and the data was too rough to analyze [18]; another one was excluded because it did not involve the indexes we were concerned about [19]. Later, two more studies were included: one in Chinese was found in the local science journal [20]; another one was found in the cited references of other articles [21]. At last a total of 11 articles were included and analyzed [20]–[30] (Table 1; Figure 1). Among them, nine were available as full-text paper and two were published in abstract form only [26], [29].

**Table 1:** Studies comparing early EN with late EN or TPN.

First author	Published year	EN started within admission	Severity of AP	Cases	Study design	Control group	EN route
Wu DC [20]	2008	48h	SAP	43	RCT	Late EN	NG
Petrov MS [21]	2006	24h	pSAP	69	RCT	TPN	NJ
Eckerwall GE [22]	2006	24h	pSAP	48	RCT	TPN	NG
Gupta R [23]	2003	24h	pSAP	17	RCT	TPN	NJ
Olah A [24]	2002	48h	AP	89	RCT	TPN	NJ
Qin HL [25]	2008	48h	SAP	74	RCT	TPN	NJ
Bakker OJ [26]	2009	48h	pSAP	296	retrospective	Late EN	NJ
McClave SA [27]	1997	48h	MAP	32	RCT	TPN	NJ
Kalfarentzos F [28]	1997	48h	SAP	38	RCT	TPN	NJ
Olah A [29]	1996	24h	AP	38	RCT	TPN	NJ
Vieira JP [30]	2010	24–48h	SAP	31	retrospective	TPN	NJ

EN, enteral nutrition; TPN, total parenteral nutrition; AP, acute pancreatitis; MAP, mild AP; SAP, severe AP; pSAP, predicted SAP; RCT, randomized comparative trial; NJ, nasojejunal feeding; NG, nasogastric feeding.

### Quality Assessment for Included Studies

Among nine included RCTs, six studies provided detailed information on the randomization techniques applied. Four generated randomization by computer programs [21], [22], [25], [28]. And one by drawing lots [23]. Olah A, et al randomized patients by their birth days and inadequately concealed allocation [29]. Allocation concealment was unclear in four studies [20], [21], [24], [27]. Only Qin HL, et al applied single-blind techniques [25]. McClave SA, et al terminated the study at the sample size less than the planned one [27]. Sample size was much too small in the study of Gupta R, et al [23]. A summary of the methodological domain assessments for each included RCTs was shown in Table 2. Two retrospective comparative trials were also included. One of them was conducted by Bakker OJ, et al and it was a large-scale and multicenter study directly comparing EN within 48 hours of admission with the late one. However, the time between admission and start of EN was at the discretion of physicians so that two groups were retrospectively defined, namely, early EN group and late EN group [26]. The other study performed by Vieira JP, et al revealed that, TPN was started within 48 hours of admission before 1999, but after 1999, EN was administered instead. This change was in conformity with the development of the artificial nutrition support [30]. Despite of its undeniable limitation of retrospection, the data report was detailed, clear, and precise.

**Table 2:** Risk of bias summary in RCTs.

First author	Adequate sequence generation?	Adequate allocation concealment?	Blinding?	Incomplete outcome data adequately addressed?	Free of selective reporting?	Free of other bias?
Wu DC [20]	?	?	−	+	+	?
Petrov MS [21]	+	?	−	+	+	+
Eckerwall GE [22]	+	+	−	+	+	+
Gupta R [23]	+	+	−	+	+	−
Olah A [24]	?	?	−	+	+	?
Qin HL [25]	+	+	+	+	+	+
McClave SA [27]	?	?	−	+	+	−
Kalfarentzos F [28]	+	+	−	+	+	?
Olah A [29]	−	−	−	+	+	+

Review authors' judgments about each risk of bias item for each included study. + is “yes”, − is “no”, ? is “unclear”.

### Effect of Early EN on all the Infection Complications of AP as a Whole

**Figure 2:**
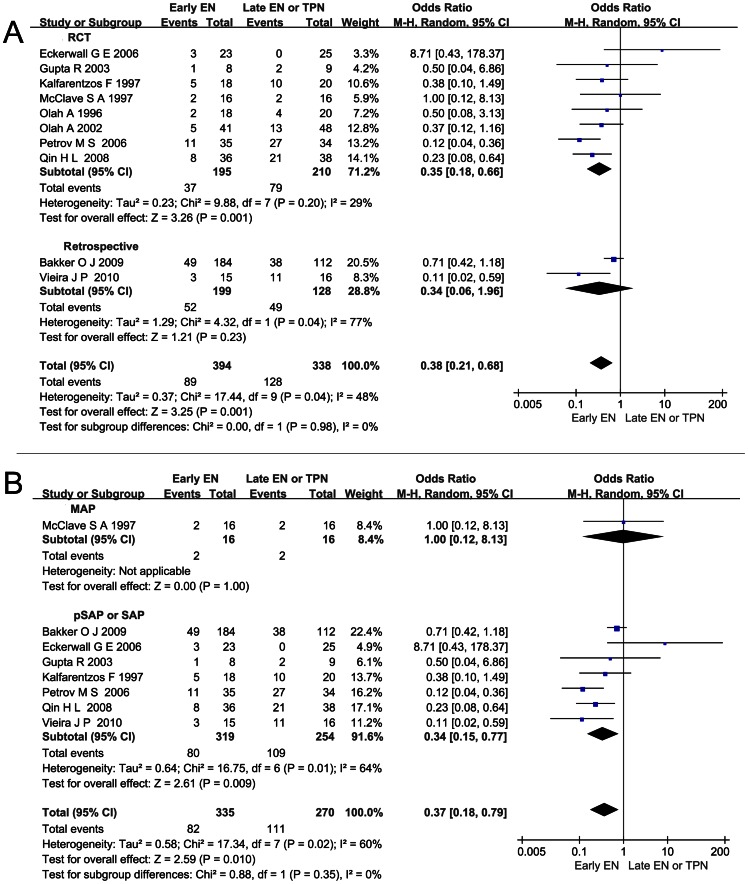
Effect of early EN on all the infection complications of AP as a whole. (A) Forest plot (Random-effect model) showing the effect of early EN on all the infection complications of AP as a whole and the sensitivity analysis subtotaling the plots by RCTs vs. retrospective studies. (B) Forest plot (Random-effect model) of the stratified study conducted based on the severity of AP.

In a pooled analysis of ten studies, we found that early EN significantly reduced all the infection complications as a whole compared with late EN or TPN (OR 0.38; 95%CI 0.21–0.68, *P*<0.05) but there was significant heterogeneity across all the studies (*I* ^2^ = 48%, *P*<0.10; Figure 2A). The severity of AP was not stated in one study conducted by Olah A, et al [24]. And in another study conducted by Olah A, et al, both MAP and SAP patients were recruited, but the incidences of infection complications were not reported respectively, while just the total number of the cases was reported [29]. Hence, neither of these studies was fit for the stratified study. Finally, eight studies were included in the stratified study. Among them, seven articles were stratified into the pSAP or SAP sub-group, showing a significant reduction in all the infections with a smaller OR (0.34; 95%CI 0.15–0.77, *P*<0.05; Figure 2B) and significant heterogeneity was detected (*I* ^2^ = 64%, *P*<0.10). But there was no subgroup difference between MAP subgroup and pSAP or SAP subgroup (*P* = 0.35).

### Effect of Early EN on Catheter-related Septic Complications of AP

**Figure 3:**
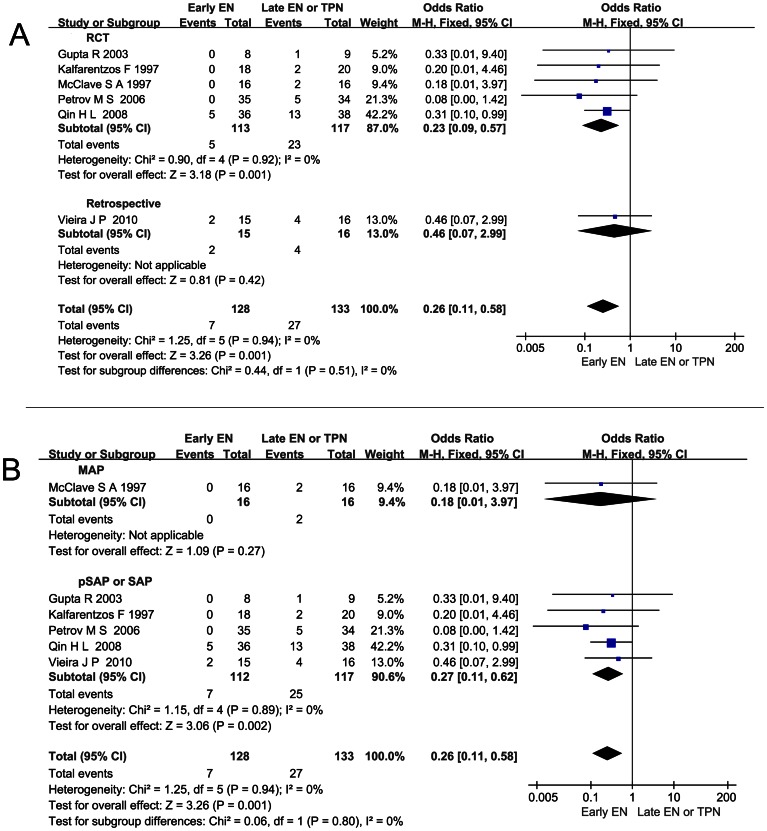
Effect of early EN on catheter-related septic complications of AP. (A) Forest plot (Fixed-effect model) showing the effect of early EN on catheter-related septic complications of AP and the sensitivity analysis subtotaling the plots by RCTs vs. retrospective studies. (B) Forest plot (Fixed-effect model) of the stratified study conducted based on the severity of AP.

In a pooled analysis of six studies observing the catheter-related septic complications, a significant reduction was observed in the early EN group compared with late EN or TPN group (OR 0.26; 95%CI 0.11–0.58, *P*<0.05). There was no significant heterogeneity across all the studies (*I* ^2^ = 0%, *P* = 0.94; Figure 3A). In the stratified study, five articles were included in the pSAP or SAP sub-group. A significant reduction was also detected (OR 0.27; 95%CI 0.11–0.62, *P*<0.05; Figure 3B) and there was no significant heterogeneity either (*I* ^2^ = 0%, *P* = 0.89). No subgroup difference between MAP subgroup and pSAP or SAP subgroup was observed (*P* = 0.80).

### Effect of Early EN on Pancreatic Infection Complications of AP

**Figure 4:**
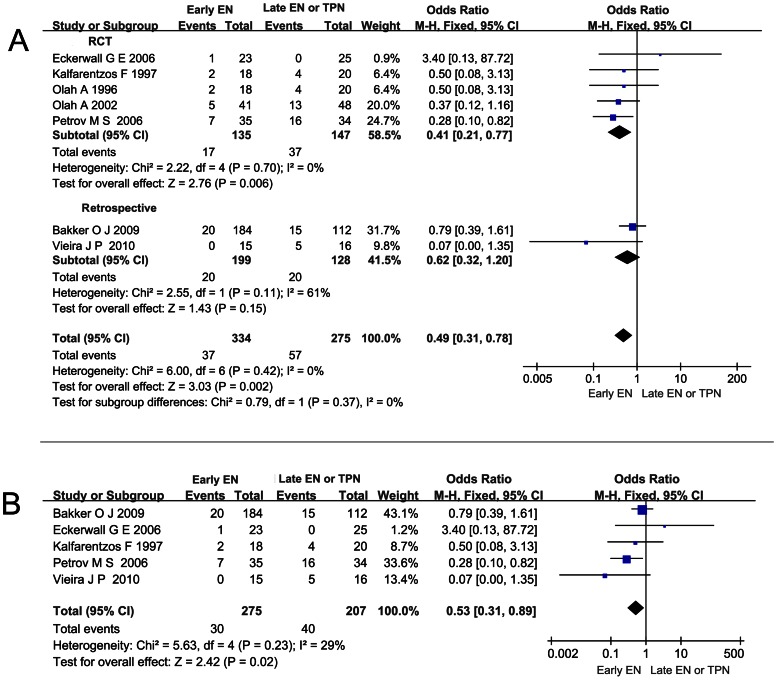
Effect of early EN on pancreatic infection complications of AP. (A) Forest plot (Fixed-effect model) showing the effect of early EN on pancreatic infection complications of AP and the sensitivity analysis subtotaling the plots by RCTs vs. retrospective studies. (B) Forest plot (Fixed-effect model) of a selection of five articles recruiting patients of pSAP or SAP.

A pooled analysis of seven articles observing the pancreatic infection complications revealed a significant reduction in the early EN group compared with late EN or TPN group (OR 0.49; 95%CI 0.31–0.78, *P*<0.05). There was no significant heterogeneity across all the studies (*I* ^2^ = 0%, *P* = 0.42; Figure 4A). As was mentioned above, two articles were not fit for the stratified study [24], [29], leaving five articles on pSAP or SAP pooled as a selection of study. A significant reduction was also detected (OR 0.53; 95%CI 0.31–0.89, *P*<0.05) and there was no significant heterogeneity either (*I* ^2^ = 29%, *P* = 0.23; Figure 4B).

### Effect of Early EN on the Incidence of Hyperglycemia as a Complication of AP

**Figure 5:**
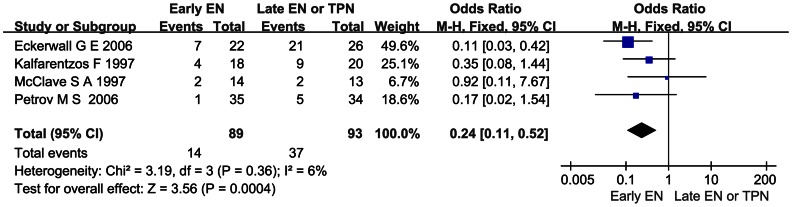
Forest plot showing the effect of early EN on the incidence of hyperglycemia in AP. Fixed-effect model was applied.

Four RCTs reporting the incidence of hyperglycemia as a complication of AP were pooled. A significant reduction was detected when comparing early EN with late EN or TPN (OR 0.24; 95%CI 0.11–0.52, *P*<0.05) and there was no significant heterogeneity across all the studies (*I* ^2^ = 6%, *P* = 0.36; Figure 5).

### Effect of Early EN on the Incidence of Pulmonary Complication in AP

Pulmonary complication, the most frequent non-pancreatic complication, was reported by eight studies. In the pooled analysis, no significant difference was detected when comparing early EN with late EN or TPN (OR 1.03; 95%CI 0.46–2.31, *P* = 0.94) and there was significant heterogeneity across all the studies (*I* ^2^ = 52%, *P*<0.10) [21]–[23], [25]–[28], [30]. Seven articles were stratified into pSAP or SAP sub-group. No significant reduction was observed either (OR 1.03; 95%CI 0.42–2.55, *P* = 0.95) with significant heterogeneity (*I* ^2^ = 59%, *P*<0.10) [21]–[23], [25], [26], [28], [30]. But there was no subgroup difference between MAP subgroup and pSAP or SAP subgroup (*P* = 0.98).

### Effect of Early EN on the Incidence of OF in pSAP or SAP

**Figure 6:**
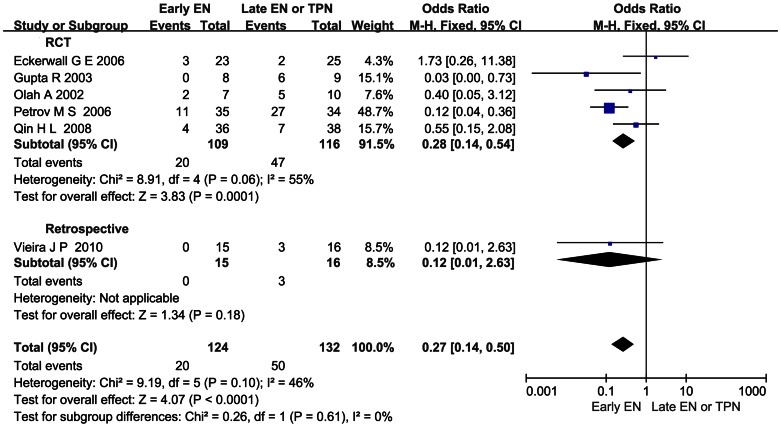
Forest plot showing the effect of early EN on the OF rate in AP. All the patients with OF were classified as pSAP or SAP previously. Fixed-effect model was applied and the sensitivity analysis subtotaling the plots by RCTs vs. retrospective studies was conducted.

Six studies reported the incidence of OF. All the patients who developed OF were classified as pSAP or SAP previously. No case of OF was reported among the MAP patients. In the pooled analysis, a significant reduction of the OF rate was detected when comparing early EN with late EN or TPN (OR 0.27; 95%CI 0.14–0.50, *P*<0.05; Figure 6). No significant heterogeneity (*I* ^2^ = 46%, *P* = 0.10) was observed.

### Effect of Early EN on the LOH in AP

**Figure 7:**
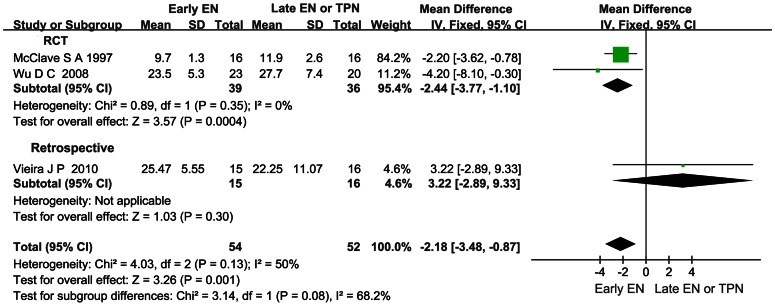
Forest plot showing the effect of early EN on the LOH in AP. Fixed-effect model was applied and the sensitivity analysis subtotaling the plots by RCTs vs. retrospective studies was conducted.

Three articles observed the LOH in AP. In the pooled analysis, a significant reduction was detected when comparing early EN with late EN or TPN (mean difference −2.18; 95%CI −3.48–(−0.87); P<0.05) and there was no significant heterogeneity across all the studies (*I* ^2^ = 50%, *P* = 0.13; Figure 7).

### Effect of Early EN on the Mortality in AP

**Figure 8:**
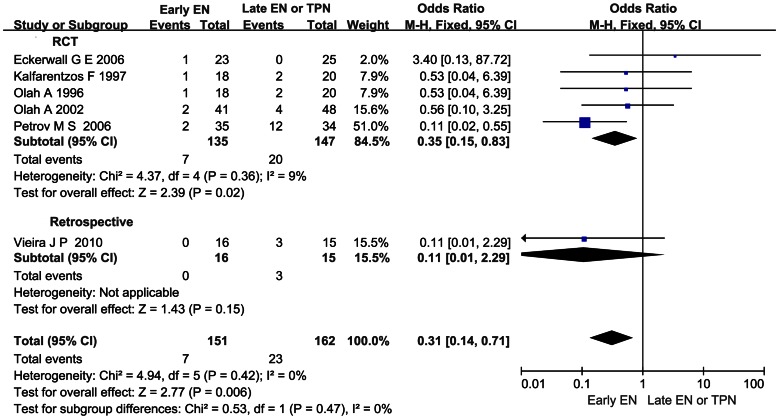
Forest plot showing the effect of early EN on the mortality in AP. Fixed-effect model was applied and sensitivity analysis subtotaling the plots by RCTs vs. retrospective studies was conducted.

Six articles observed the mortality in AP. In the pooled analysis, a significant reduction was detected when comparing the effect of early EN with that of late EN or TPN (OR 0.31; 95%CI 0.14–0.71, *P*<0.05; Figure 8). There was no significant heterogeneity across all the studies (*I* ^2^ = 0%, *P* = 0.42).

### Summary of Methodological Quality and Risk of Bias

Since two retrospective studies were pooled with the RCTs in this analysis, sensitivity analysis to subtotal the plots by RCTs vs. retrospective studies was conducted (Table 3). In all the statistical analyses of clinical outcomes except those about all the infection complications, heterogeneity did not turn significant when retrospective studies were pooled with the RCTs. (Figure 2A). And no significant differences of OR between RCTs and retrospective studies were detected (all *P*>0.05; Table 3; Fiuge2A, 3A. 4A, 6, 7, 8). The potential publication bias on the association of each clinical outcome and early EN was assessed and demonstrated by the Begg’s and the Egger’s test quantitatively. No significant publication bias was found (all *Pr* or *P*>0.05; Table 4).

**Table 3:** Sensitivity analysis subtotaling the plots by RCTs vs. retrospective studies.

Clinical outcome	RCTs			Retrospective studies			Total			Subgroup differences*P* =
	OR	[95%CI]	Heterogeneity (*p* = )	OR	[95%CI]	Heterogeneity (*p* = )	OR	[95%CI]	Heterogeneity (*p* = )	
All the infection Complications	0.35	[0.18,0.66]	0.20	0.34	[0.06,1.96]	0.04	0.38	[0.21,0.68]	0.04	0.98
Catheter-related septic complications	0.23	[0.09,0.57]	0.92	0.46	[0.07,2.99]	/*	0.26	[0.11,0.58]	0.94	0.51
Pancreatic infection complications	0.41	[0.21,0.77]	0.70	0.62	[0.32,1.20]	0.11	0.49	[0.31,0.78]	0.42	0.37
Hyperglycemia&	0.24	[0.11,0.52]	0.36	/	/	/	0.24	[0.11,0.52]	0.36	/
Pulmonary complications	1.09	[0.47,2.57]	0.03	0.33	[0.01,8.33]	/*	1.03	[0.46,1.31]	0.04	0.49
OF	0.28	[0.14,0.54]	0.06	0.12	[0.01,2.63]	/*	0.27	[0.14,0.50]	0.10	0.61
the LOH	−2.44	[−3.77,−1.10]	0.35	3.22	[−2.89,9.33]	/*	−2.18	[−3.48,−0.87]	0.13	0.08
Mortality	0.35	[0.15,0.83]	0.36	0.11	[0.01,2.99]	/*	0.31	[0.14,0.71]	0.42	0.47

& All the studies that observed the incidence of hyperglycemia were RCTs.

* Because only one retrospective study was involved, the assessment of heterogeneity was not applicable.

**Table 4:** Publication bias on the association of each clinical outcome and early EN.

Clinical outcome	Begg’s test (*Pr*>|z| = )	Egger’s test (*P*> |t| = )
All the infection complications	0.251	0.797
Catheter-related septic complications	1.00	0.153
Pancreatic infection complications	1.00	0.724
Hyperglycemia	1.00	0.874
Pulmonary complications	0.902	0.052
OF	0.707	0.932
the LOH	1.00	0.959
Mortality	0.573	0.292

## Discussion

AP causes local and systemic complications, leading to high catabolic, hypermetabolic, and hyperdynamic stress states. It has been drawn attention for decades and many researches on nutrition support have been conducted. As a result of the proven advantages of EN compared with TPN, EN is increasingly advocated in clinic guidelines [31], [32]. But the advantage of very early EN without “gut rest” has not been well established. We intended to find out whether the early initiation of EN without “gut rest” is feasible and improves the clinical outcomes. And the reason why we chose 48 hours as the time window was that the starvation period was so short that it was considered to have no “gut rest” and this “cut-off” time point was recommended by the ASPEN (American Society for Parenteral and Enteral Nutrition) guideline [33]. So we carefully conducted the selection for the uniformity and only the studies strictly in conformity with the time window (48 hours within admission) were included. Studies, which defined early EN as “within 72 hour of admission”, “after 48 hours of enrollment” or “within 72 hours of onset” and so on, were excluded. Our study indicates that, above all, early EN is practical in AP and even in SAP. It was reported by the included studies that no patients had to drop out from the early EN group or turn to TPN support because of lethal event or failure to fulfill the nutrition demand. Only Gupta R, et al reported in their studies that two patients in the early EN group required temporary reduction in the volume of their EN because of gastrointestinal symptoms [23].

Infection complications are the major factors contributing to the poor outcome of AP. Researchers assumed that early EN could lower the infection complications in two ways: (1) EN could reduce the catheter-related infection associated with TPN in which central venous catheter was used and it has been proven by a clinic research [34]; (2) EN could help maintain the integrity of the intestinal mucosa barrier and reduce bacteria translocation from small intestine which was observed within 24 hours in the natural course of AP in the rat models [35], [36]. The current pooled analysis proves that early EN significantly reduces the risk of all the infections as a whole, the catheter-related infection, and the pancreatic infection (Figure 2A, 3A, 4A). So it’s rational to count on the effect of early EN in reducing the infections in both ways.

Patients classified as SAP were found to have higher intestinal permeability, serum endotoxin level, and cytokine level [37]. More remote organ involvement or even OF caused by SIRS might occur. And in all the studies we included, OF was only reported in the population of SAP. Since the integral gut mucosa could not only lower the bacteria translocation but also decrease the toxins, oxidative stress, and inflammation factors release, maintaining the gut barrier in early phase to hinder the harm in pSAP or SAP was emphasized [38], [39]. Our stratified analysis based on the severity of AP confirms that early EN shows a stronger protective power against all the infection complications and also significantly reduces the risk of catheter-related septic complications and pancreatic infection complications (Figure 2B, 3B, 4B). As to OF, the particular complication of pSAP or SAP, was also reduced significantly when early EN was administered (Figure 6). Additionally, in the early EN group, Qin HL, et al found that CRP level was significantly lower and Wu DC, et al reported that shorter time was taken for the raised CRP level to return to normal [20], [25].

Infection and OF continue to cause death (the overall mortality rate is approximately 10%) despite immense improvements in supportive, radiologic, and surgical therapy in AP [40]. Reduction of infection and OF rate in early EN group could bring about reduction of the LOH and the mortality. And it was found to be significant in our analysis (Figure 7, 8). The findings were consistent with the systematic review conducted by Petrov MS, et al [41]. They demonstrated that EN initiated within 48 hours of admission, in comparison with late EN or TPN, resulted in a statistically significant reduction in the risks of multiple OF, pancreatic infections complications, and the mortality in AP. And we included five more trials and added stratified analyses in our meta-analysis for further study [20], [25], [26], [29], [30].

Pulmonary complication is the most common non-pancreatic complication, which is caused by various mechanisms. Acute respiratory distress syndrome may be associated with a complex cascade of events including inflammation that start with early acinar cell damage in AP [42]. Besides, it was observed in rat model that dexamethasone could down-regulate the inflammatory mediators in the lung but failed to hinder the lung injury or complication, which might be attributed to the leukocyte recruitment [43]. So we assumed that early EN which could reduce the release of inflammatory mediators from the gut was not enough to reduce pulmonary complications. And this was proven by our analysis that the incidence of pulmonary complications did not differ between early EN group and late EN or TPN group. More studies on effective strategies to reduce the pulmonary complications are needed.

Hyperglycemia is the major metabolic complication of AP, especially of SAP. A recent trial in critical illness demonstrated that, the euglycemic state, namely glucose level 80–140 mg/dL, reduced the incidence of polyneuropathy and duration of ventilator dependency in medical intensive care unit [44]. It was recommended by practical guidelines to control glucose level to <150 and absolutely <180 mg/dL in critical illnesses [45]. The present study reveals that hyperglycemia was significantly reduced in the early EN group (Figure 5). Though the effect of the restoration of normoglycemia in AP has not been studied, considering that AP, especially SAP, is a critical illness, this concept could also apply to AP and might improve the clinic outcome.

### Limitations

There are several limitations in our study. Firstly, not all the articles included were RCTs. So we conducted a sensitivity analysis to subtotal the plots by RCT vs. retrospective studies. No significant differences of OR between RCTs and retrospective studies were detected and the retrospective studies only brought heterogeneity in one of the eight clinical outcomes (Table 3; Fiuge2A, 3A. 4A, 6, 7, 8). So the same conclusion could be drawn no matter whether RCTs were pooled with the retrospective studies or not. Hence, the retrospective studies included here did not pull things offline. Secondly, because of the different feeding routes, it’s hard to conduct double-blinded RCTs. But the clinical indexes we chose, such as the incidence of infection complications, were seldom affected by subjective feelings. Last but not least, the present analysis proves definitely that EN in the very early phase of AP without “gut rest” is superior to TPN which makes gut totally rest. But is EN started the earlier the better? The evidence for it is insufficient, because the control group in the studies we meta-analyzed was a mixed group mainly composed of TPN. And hyperglycemia is a well-known complication of TPN, thus the conclusion that early EN has advantage in controlling hyperglycemia is not robust enough. However, two of the included studies did compare early EN with late EN directly and indicated that early EN was superior to the late one [20], [26]. But one of them had a rather small sample size and the other was a retrospective study (Table 2). The systematic review conducted by Petrov MS, et al demonstrated that in comparison with TPN, EN within 48 hours could reduce complications and EN initiated after 48 hours did not result in significant reduction of complications [41]. But this was still not enough to prove the importance of the timing of EN. All of these findings reveal that a large-scale, well-organized, and adequately powered RCT that directly compares early EN with late EN, such as the PYTHON trial, is necessary [18].

### Conclusions

This analysis reveals that EN initiated within 48 hours of admission improves the clinical outcomes of AP by reducing the risk of infections, OF, hyperglycemia, death, and shortening the LOH as well as in pSAP or SAP. So, even if in the very early phase of AP, when artificial nutrition is taken into consideration, EN, rather than TPN, is recommended.

## Supporting Information

Supplement S1 **PRISMA Checklist.**
(DOC)
